# Predictors of Delayed Entry into Medical Care of Children Diagnosed with HIV Infection: Data from an HIV Cohort Study in India

**DOI:** 10.1155/2013/737620

**Published:** 2013-11-14

**Authors:** Gerardo Alvarez-Uria, Praveen Kumar Naik, Manoranjan Midde, Raghavakalyan Pakam

**Affiliations:** Department of Infectious Diseases, Bathalapalli Rural Development Trust Hospital, Kadiri Road, Bathalapalli, Anantapur District, Andhra Pradesh 515661, India

## Abstract

Data about the attrition before entry into care of children diagnosed with HIV in low- or middle-income countries are scarce. The aim of this study is to describe the attrition before engagement in HIV medical care in 523 children who were diagnosed with HIV from 2007 to 2012 in a cohort study in India. The cumulative incidence of children who entered into care was 87.2% at one year, but most children who did not enter into care within one year were lost to followup. The mortality before entry into care was low (1.3% at one year) and concentrated during the first three months after HIV diagnosis. Factors associated with delayed entry into care were being diagnosed after mother's HIV diagnosis, belonging to scheduled castes, age <18 months, female gender, and living >90 minutes from the HIV centre. Children whose parents were alive and were living in a rented house were at a higher risk of delayed entry into care than those who were living in an owned house. The results of this study can be used to improve the linkage between HIV testing and HIV care of children diagnosed with HIV in India.

## 1. Introduction

In low- and middle-income countries, children who are diagnosed with HIV are referred to HIV medical centres, commonly called antiretroviral therapy (ART) centres, where they can receive specialized care and initiate ART [1]. Children who do not enter into care are at a high risk of death and HIV-related morbidities [2–4]. Studies on adults have shown that 20–30% of patients who are diagnosed with HIV do not enter into care [5, 6]. However, data about the attrition of HIV-infected children before entering into medical care are scarce [7].

India has the highest burden of paediatric HIV in Asia, and 14,500 children acquire HIV every year [8]. According to governmental data, there are 145,000 children living with HIV in India, but only 112,385 (77.5%) of them had been registered in ART centres by December 2012 [8]. The objective of this study is to describe the proportion of children who do not enter into care after being diagnosed with HIV in a cohort study in India. In particular, we aimed to find predictors of delayed entry into care, which could help HIV programmes to design interventions aimed at increasing the number of HIV-infected children entering into care in India.

## 2. Methods

### 2.1. Setting and Design

The study was performed in Anantapur, a district situated in the south border of Andhra Pradesh, India. Anantapur has a population of approximately four million people, and 72% of them live in rural areas [9]. The HIV epidemic in Anantapur is largely driven by heterosexual transmission and it is characterized by poor socioeconomic conditions and high levels of illiteracy [10]. Rural Development Trust (RDT) is a nongovernmental organization that has three hospitals in the district. RDT provides medical care to HIV-infected people free of cost, including medicines, consultations, and hospital admission charges. Patients who are diagnosed with HIV are referred to an ART centre located in RDT Bathalapalli Hospital, where CD4+ lymphocyte count determinations and ART are provided free of cost [11]. The Vicente Ferrer HIV Cohort Study (VFHCS) is an open cohort study of all HIV-infected patients who have attended RDT hospitals. The characteristics of the cohort have been described in detail elsewhere [10, 12]. For this study, we selected patients who were <15 years old at the time of HIV diagnosis, living in Anantapur, and diagnosed with HIV between January 1, 2007, and December 31, 2012. The selection of patients from the database was executed on July 15, 2013. Patients who were lost to followup (LTFU) were actively searched by phone calls and home visits by outreach workers, and, in those patients who had died, relatives were asked the date of death of the patient. To assess the entry into HIV care, we calculated the time period between the diagnosis of HIV and the first CD4+ lymphocyte count determination or ART initiation, whatever occurred first. In children aged <18 months, the HIV diagnosis was performed using polymerase chain reaction virological assays [13, 14]. 

### 2.2. Definitions

Designation of the community of patients was performed by self-identification. Scheduled caste community is marginalised in the traditional Hindu caste hierarchy and, therefore, suffers social and economic exclusion and disadvantage. Scheduled tribe community is generally geographically isolated with limited economic and social contact with the rest of the population. Scheduled castes (SC) and scheduled tribes are considered socially disadvantaged communities and are supported by positive discrimination schemes operated by the Government of India. Backward castes (BC) form a collection of “intermediate” castes that were considered low in the traditional caste hierarchy, but above scheduled castes [15]. Patients were considered as living near a town when they lived in a mandal (administrative subdivision of districts in Andhra Pradesh; e.g., Anantapur District has 64 mandals) containing a town with a population of >100,000 people. In those children whose both parents were alive, parents were asked whether they lived in a rented house or in an owned house, as a marker of the economic conditions of the caregivers.

### 2.3. Statistical Analysis

Statistical analysis was performed using Stata Statistical Software (Stata Corporation, Release 11. College Station, TX, USA). To investigate predictors of delayed entry into care, time-to-event methods were used. Time was measured from HIV diagnosis to entry into care or death, whatever occurred first. Children who did not die nor enter into care were censored at the end of the follow-up period (July 15, 2013). Children who did not come to the clinics for at least 180 days after their last visit date were considered LTFU [16]. Cox regression models assume that the distribution of censoring times and the time-to-event distribution are independent of each other [17]. When studying the cumulative incidence of entry into care, a group of patients will be censored at death. However, dead children will not be able to enter into care [18]. Including these children in standard survival models may lead to an overestimation of the event of interest. Thus, multivariable analysis and estimation of the cumulative incidence of entry into care were performed using competing risk proportional hazard models with death before entry into care as a competing event [19]. These models estimate subdistribution hazard ratios (SHRs), which can be interpreted similarly to hazard ratios estimated by Cox proportional models, but they take into account the hazard of the competing event [17]. The proportional hazard assumption was assessed performing log-log survival curves based on Schoenfeld residuals [20]. Cumulative incidence of entry into care and death was estimated using the “stcompet” command in Stata [21, 22]. The study was approved by the ethical committee of the RDT Institutional Review Board.

## 3. Results

We identified 526 children from the VFHCS who were diagnosed with HIV from 2007 to 2012. Three children who were transferred to other ART centres were not included in the analysis. The study included 264 child-years, and, during the study period, nine children died and 38 were LTFU. Among children who died, the median time from HIV diagnosis to death was 1.6 months (interquartile range (IQR), 0.3–3.6) and, in those who did not enter into care, the median followup was 56 months (IQR, 44.6–66.2). The median time from HIV diagnosis to entry into care was 0.2 months (IQR, 0.03–0.9). Baseline characteristics and multivariable analysis of factors associated with entry into medical care are described in Table 1. The median age at HIV diagnosis was 60 months (IQR, 31.5–102.4); over half were female, in 98% of them, HIV was vertically transmitted, and in 92%, the HIV diagnosis was made after their mothers were diagnosed with HIV. Over half belonged to BC communities and 63% were living far from town. The majority of children were diagnosed between 2007 and 2009, and over half needed more than one hour to reach the ART centre. Near half of the children had lost one or both of their parents, and the majority of those whose parents were alive were living in a rented house. Factors associated with delayed entry into medical care were being diagnosed at earlier calendar years, being diagnosed after knowing that the mother was HIV positive, belonging to SC communities, age <18 months, female gender, and living >90 minutes from the ART centre. Those children whose parents were alive and were living in a rented house were at a higher risk of delayed entry into care than those who were living in an owned house. 

**Figure 1:**
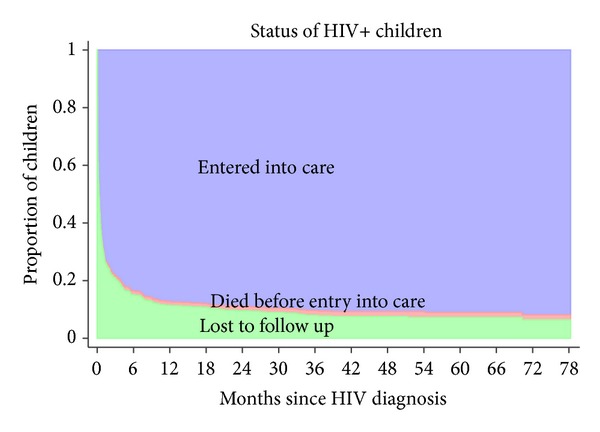
Stacked graph describing the cumulative incidence of entry into care and death after HIV diagnosis. Children diagnosed with HIV infection were considered lost to follow up until they entered into care.

A stacked graph of the status of HIV-infected children since HIV diagnosis is presented in Figure 1. The cumulative incidence of entry into care was 78.4% (95% CI, 74.6–81.7) at 3 months, 83.6% (95% CI, 80.1–86.5) at 6 months, 87.2% (95% CI, 84–89.8) at 1 year, 88.9% (95% CI, 85.9–91.3) at 2 years, 90.3% (95% CI, 87.4–92.6) at 3 years, 90.7% (95% CI, 87.9–92.9) at 4 years, 91.1% (95% CI, 88.2–93.3) at 5 years, and 91.9% (95% CI, 88.7–94.2) at 6 years. The cumulative incidence of death before entry into care was 1.1% (95% CI, 0.5–2.4) at 3 months, 1.3% (95% CI, 0.6–2.6) at 1 year, 1.5% (95% CI, 0.7–2.9) at 2 years, and 1.7% (95% CI, 0.9–3.2) at 3 years. 

## 4. Discussion 

This study shows that nearly 90% of children diagnosed with HIV enter into care within one year. This figure is similar to the ones found in studies from sub-Saharan Africa, where it was found that 78–97% of children enter into care [23–26], and this is higher than the proportion of adults who enter into care within one year of HIV diagnosis in our setting, which was 77.4% (95% CI, 76.5–78.3) in a study using the same methodology [5]. However, similar to adults in our setting [5], the majority of children who do not enter into care within one year were LTFU. These children are at a high risk of death or may engage in care only after developing opportunistic infections or other HIV-related pathologies [4, 24]. 

To our knowledge, this is one of the first studies to describe predictors of delayed entry into care in children from a resource-limited setting outside sub-Saharan Africa. Children from SC communities and those whose parents were alive and living in a rented house were less likely to enter into care. This suggests that children born in families with low socioeconomic status were more likely to enter into care late. In a qualitative study in Western Kenya, some of the mothers' reasons for not taking their children to the clinics were transport costs, food availability, time constraints due to work commitment, and unsupportive male partner [27]. In our setting, many families live in extreme poverty conditions [10], and the health of a child recently diagnosed with HIV may not be their first priority [28]. 

In contrast with studies from sub-Saharan Africa [24, 29], female children were less likely to enter into care than male children, reflecting the discrimination against female children in rural India [30]. Children diagnosed with HIV after knowing that their mothers were HIV-infected were less likely to enter into care than children who were diagnosed for other reasons. It is possible that children whose mothers' HIV status was not known attended the clinics complaining of symptoms related to their HIV infection. Therefore, searching for a cure for their symptoms might have motivated caregivers to attend ART centres. Living far from an ART centre was also associated with a delayed entry into care, which supports the current policy of the decentralization of ART centres by the Indian Government. In line with studies from sub-Saharan Africa [24, 29], children diagnosed at age <18 months were less likely to enter into care, which could be related to the high mortality observed in HIV-infected children during the first two years of life [3].

Children diagnosed with HIV at more recent calendar years were more likely to enter into care, suggesting that the linkage between HIV testing and ART centres has improved in recent years. We also observed a reduced number of children diagnosed with HIV since 2010, which could be related to the implementation of a new programme to reduce mother-to-child transmission with universal antiretroviral therapy in our district since 2008 [31]. 

The study has some limitations. We did not have information of all children diagnosed with HIV in the district, so children diagnosed in other clinics who never came to our hospital were not included in the study. Therefore, the proportion of children diagnosed with HIV who entered into care is likely to be overestimated. 

## 5. Conclusions

In our setting, the majority of children diagnosed with HIV enter into care within one year, but most children who do not enter into care within one year remain LTFU. Being diagnosed after discovering the HIV status of the mother, having a low socioeconomic status, age <18 months, female gender, and living far from the ART centre were factors associated with delayed entry into care. HIV programmes in India should consider this information to improve the linkage between HIV testing and ART centres, by offering better support to children within these risk groups. 

## Figures and Tables

**Table 1:** Baseline characteristics and multivariable analysis of factors associated with entry into care using competing risk regression of 523 children diagnosed with HIV in Anantapur, India.

	N (%)	SHR
Age		
<18 months	65 (12.43)	0.67* (0.49–0.91)
18–59 months	197 (37.67)	1 (Reference)
5–9 years	179 (34.23)	1.05 (0.85–1.29)
10–15 years	82 (15.68)	1.03 (0.80–1.33)
Gender		
Female	267 (51.05)	0.79* (0.67–0.94)
Male	256 (48.95)	1 (Reference)
Community		
OC	107 (20.46)	0.97 (0.77–1.22)
BC	270 (51.63)	1 (Reference)
SC	117 (22.37)	0.78* (0.64–0.97)
ST	29 (5.54)	1.01 (0.70–1.45)
HIV transmission		
Vertical	512 (97.9)	1.58 (0.87–2.89)
Other	11 (2.1)	1 (Reference)
Living near a town		
No	328 (62.72)	1 (Reference)
Yes	195 (37.28)	0.94 (0.78–1.13)
Year of diagnosis		
2007	109 (20.84)	1 (Reference)
2008	97 (18.55)	1.16 (0.89–1.53)
2009	109 (20.84)	1.10 (0.84–1.46)
2010	92 (17.59)	2.07* (1.63–2.63)
2011	61 (11.66)	1.55* (1.11–2.15)
2012	55 (10.52)	2.05* (1.48–2.83)
Time to ART centre		
≤30 min	139 (26.58)	1 (Reference)
31–60 min	96 (18.36)	1.03 (0.81–1.31)
61–90 min	116 (22.18)	0.93 (0.74–1.18)
>90 min	172 (32.89)	0.80* (0.64–0.99)
Status of parents		
Alive, rented house	170 (32.5)	0.59* (0.46–0.76)
Alive, owned house	117 (22.37)	1 (Reference)
Father died	119 (22.75)	0.95 (0.75–1.22)
Mother died	51 (9.75)	0.81 (0.58–1.12)
Both died	66 (12.62)	0.83 (0.61–1.14)
Reason for HIV testing		
HIV+ mother	481 (91.97)	0.62* (0.48–0.81)
Others	42 (8.03)	1 (Reference)

*P value < 0.05. ART: antiretroviral therapy; BC: backward castes; OC: other castes; SHR: adjusted subdistribution hazard ratio; SC: scheduled castes; ST: scheduled tribes.

## References

[1] National AIDS Control Organisation (2006). *Guidelines for HIV Care and Treatment in Infants and Children*.

[2] Spira R, Lepage P, Msellati P (1999). Natural history of human immunodeficiency virus type 1 infection in children: a five-year prospective study in Rwanda. Mother-to-Child HIV-1 Transmission Study Group. *Pediatrics*.

[3] Newell M-L, Coovadia H, Cortina-Borja M, Rollins N, Gaillard P, Dabis F (2004). Mortality of infected and uninfected infants born to HIV-infected mothers in Africa: a pooled analysis. *The Lancet*.

[4] Edmonds A, Yotebieng M, Lusiama J (2011). The effect of highly active antiretroviral therapy on the survival of HIV-infected children in a resource-deprived setting: a cohort study. *PLoS Medicine*.

[5] Alvarez-Uria G (2013). Factors associated with delayed entry into HIV medical care after HIV diagnosis in a resource-limited setting: data from a cohort study in India. *PeerJ*.

[6] Mugglin C, Estill J, Wandeler G, Bender N, Egger M, Gsponer T (2012). Loss to programme between HIV diagnosis and initiation of antiretroviral therapy in sub-Saharan Africa: systematic review and meta-analysis. *Tropical Medicine & International Health*.

[7] Mugglin C, Wandeler G, Estill J (2013). Retention in care of HIV-infected children from HIV test to start of antiretroviral therapy: systematic review. *PLoS ONE*.

[8] National AIDS Control Organisation (2013). HIV estimates 2012.

[9] Office of The Registrar General & Census Commissioner (2011). *Census of India*.

[10] Alvarez-Uria G, Midde M, Pakam R, Naik PK (2012). Gender differences, routes of transmission, socio-demographic characteristics and prevalence of HIV related infections of adults and children in an HIV cohort from a rural district of India. *Infectious Disease Reports*.

[11] Alvarez-Uria G, Reddy R, Reddy S, Naik PK, Midde M (2012). Evaluation of a low-cost strategy for enumerating CD4 lymphocyte absolute count and percentage using the FACSCalibur flow cytometer in HIV-infected patients from a resource-limited setting. *ISRN Aids*.

[12] Alvarez-Uria G, Midde M, Pakam R, Kannan S, Bachu L, Naik PK (2012). Factors associated with late presentation of HIV and estimation of antiretroviral treatment need according to CD4 lymphocyte count in a resource-limited setting: data from an HIV cohort study in India. *Interdisciplinary Perspectives on Infectious Diseases*.

[13] Alvarez-Uria G, Azcona JM, Reddy S, Midde M, Naik PK, Reddy NR (2012). Point of care testing of HIV in children younger than 18 months with three different HIV virological assays. Experience from a district hospital in a resource-limited setting. *Microbiology Research*.

[14] Alvarez-Uria G, Naik PK, Midde M, Kannan S, Reddy R (2012). False negative HIV antibody test in HIV infected children who receive early antiretroviral treatment in a resource-limited setting. *Infectious Disease Reports*.

[15] Alvarez-Uria G, Midde M, Naik PK (2012). Socio-demographic risk factors associated with HIV infection in patients seeking medical advice in a rural hospital of India. *Journal of Public Health Research*.

[16] Chi BH, Yiannoutsos CT, Westfall AO (2011). Universal definition of loss to follow-up in HIV treatment programs: a statistical analysis of 111 facilities in Africa, Asia, and Latin America. *PLoS Medicine*.

[17] Ingle SM, May M, Uebel K (2010). Outcomes in patients waiting for antiretroviral treatment in the Free State Province, South Africa: prospective linkage study. *Aids*.

[18] Geng EH, Bwana MB, Muyindike W (2013). Failure to initiate antiretroviral therapy, loss to follow-up and mortality among HIV-infected patients during the pre-ART period in Uganda. *Journal of Acquired Immune Deficiency Syndromes*.

[19] Fine JP, Gray RJ (1999). A proportional hazards model for the subdistribution of a competing risk. *Journal of the American Statistical Association*.

[20] Kleinbaum DG, Klein M (2005). *Survival Analysis, a Self-Learning Text*.

[21] Coviello V, Boggess M (2004). Cumulative incidence estimation in the presence of competing risks. *Stata Journal*.

[22] Cleves MA, Gould WW, Gutierrez RG (2008). *An Introduction to Survival Analysis Using Stata*.

[23] Nyandiko WM, Mwangi A, Ayaya SO (2009). Characteristics of HIV-infected children seen in Western Kenya. *East African Medical Journal*.

[24] Anaky M-F, Duvignac J, Wemin L (2010). Scaling up antiretroviral therapy for HIV-infected children in Côte d’Ivoire: determinants of survival and loss to programme. *Bulletin of the World Health Organization*.

[25] Leyenaar JK, Novosad PM, Ferrer KT (2010). Early clinical outcomes in children enrolled in human immunodeficiency virus infection care and treatment in lesotho. *Pediatric Infectious Disease Journal*.

[26] Sutcliffe CG, van Dijk JH, Bolton-Moore C, Cotham M, Tambatamba B, Moss WJ (2010). Differences in presentation, treatment initiation, and response among children infected with human immunodeficiency virus in urban and rural Zambia. *Pediatric Infectious Disease Journal*.

[27] Wachira J, Middlestadt SE, Vreeman R (2012). Factors underlying taking a child to HIV care: implications for reducing loss to follow-up among HIV-infected and -exposed children. *Journal of Social Aspects of HIV/AIDS*.

[28] Dhillon PK, Jeemon P, Arora NK, Mathur P, Maskey M, Sukirna RD (2012). Status of epidemiology in the WHO South-East Asia region: burden of disease, determinants of health and epidemiological research, workforce and training capacity. *International Journal of Epidemiology*.

[29] Okomo U, Togun T, Oko F, Peterson K, Jaye A (2012). Mortality and loss to programme before antiretroviral therapy among HIV-infected children eligible for treatment in The Gambia, West Africa. *AIDS Res Ther*.

[30] Bandyopadhyay M (2003). Missing girls and son preference in rural India: looking beyond popular myth. *Health Care for Women International*.

[31] Alvarez-Uria G, Midde M, Pakam R, Bachu L, Naik PK (2012). Effect of formula feeding and breastfeeding on child growth, infant mortality, and HIV transmission in children born to HIV-infected pregnant women who received triple antiretroviral therapy in a resource-limited setting: data from an HIV cohort study in India. *ISRN Pediatrics*.

